# Clinical aspects of binge eating disorder: A cross-sectional mixed-methods study of binge eating disorder experts' perspectives

**DOI:** 10.3389/fpsyt.2022.1087165

**Published:** 2023-02-14

**Authors:** Brenna Bray, Adam Sadowski, Chris Bray, Ryan Bradley, Heather Zwickey

**Affiliations:** ^1^Helfgott Research Institute, National University of Natural Medicine, Portland, OR, United States; ^2^Wilder Research Division, Amherst H. Wilder Foundation, Saint Paul, MN, United States; ^3^Herbert Wertheim School of Public Health, University of California, San Diego, San Diego, CA, United States

**Keywords:** binge eating disorder, binge eating, eating disorder, obesity, restriction, diet, emotion regulation, diagnostic criteria

## Abstract

**Introduction:**

Research on binge eating disorder continues to evolve and advance our understanding of recurrent binge eating.

**Methods:**

This mixed-methods, cross-sectional survey aimed to collect information from experts in the field about clinical aspects of adult binge eating disorder pathology. Fourteen experts in binge eating disorder research and clinical care were identified based on receipt of relevant federal funding, PubMed-indexed publications, active practice in the field, leadership in relevant societies, and/or clinical and popular press distinction. Anonymously recorded semi-structured interviews were analyzed by ≥2 investigators using reflexive thematic analysis and quantification.

**Results:**

Identified themes included: (1) obesity (100%); (2) intentional/voluntary or unintentional/involuntary food/eating restriction (100%); (3) negative affect, emotional dysregulation, and negative urgency (100%); (4) diagnostic heterogeneity and validity (71%); (5) paradigm shifts in understanding binge eating disorder (29%); and (6) research gaps/future directives (29%).

**Discussion:**

Overall, experts call for a better understanding of the relationship between binge eating disorder and obesity, including a need for clarification around the extent to which the two health issues are separate vs. related/overlapping. Experts also commonly endorse food/eating restriction and emotion dysregulation as important components of binge eating disorder pathology, which aligns with two common models of binge eating disorder conceptualization (e.g., dietary restraint theory and emotion/affect regulation theory). A few experts spontaneously identified several paradigm shifts in our understanding of who can have an eating disorder (beyond the anorexi-centric “thin, White, affluent, *cis*-gendered neurotypical female” stereotype), and the various factors that can drive binge eating. Experts also identified several areas where classification issues may warrant future research. Overall, these results highlight the continual advancement of the field to better understand adult binge eating disorder as an autonomous eating disorder diagnosis.

## Introduction

Binge eating disorder (discrete rapid consumption of objectively large amounts of food associated with loss of control and distress without compensatory behaviors) became a formally recognized autonomous eating disorder diagnosis with the publication of the *Diagnostic and Statistical Manual of Mental Disorders, 5th Edition* (DSM-V) in 2013 (1). It was previously classified in the DSM-IV as eating disorder not otherwise specified (ED-NOS) (2). While research and literature on binge eating disorder have been growing, historically there has been greater understanding and awareness of anorexia nervosa and bulimia nervosa, and less so of binge eating disorder. However, the literature continues to evolve and advance our understanding of recurrent binge eating.

Historically, there is a tendency to view binge eating disorder as resulting from overevaluation of body weight/shape/size leading to food/eating restriction and subsequent binge eating (e.g., transdiagnostic-, dietary restraint-, and dual pathway models) (3–7). However, several alternative conceptualizations of binge eating disorder have gained attention in recent years (4).

Emotion/affect regulation models are perhaps the most widely supported and accepted in the field, along with dietary restraint models (4). These models center around the view that negative emotions, moods, or affective experiences can prompt binge eating, which can become negatively reinforced by providing temporary relief from the associated discomfort (4). In this way, it is believed binge eating can become a maladaptive emotion regulation/coping strategy resulting from lack of more adaptative tools. In these models, the aversive experiences that drive binge eating often include distress (unhappiness, pain, and/or suffering affecting the mind or body) and negative affect (the subjective experience of a cluster of negative emotional states that include anxiety, depression, stress, sadness, worry, guilt, shame, anger, and envy), which can result in negative urgency (an impulsive inclination to engage in risky or unhealthy behaviors when in a state of poor emotion regulation) (8–12). These models are strongly supported in the literature (4, 8–10) and—along with dietary restraint—represent commonly overlapping concepts across various conceptualizations of binge eating disorder (e.g., dual pathway models, escape/disassociation models, ICAT models, interpersonal models, and transdiagnostic models) (4).

The issue of obesity also remains a point of contention in the field. Literature demonstrates binge eating disorder has a 40–70% incidence of lifetime obesity (13–15) and obesity has a ≤ 47% prevalence of binge eating disorder (16). However, there remains a need for updated information on the extent to which binge eating disorder and weight issues are separate/related/overlapping.

Negative health implications associated with obesity (e.g., cardiometabolic syndrome) highlight another important question of the extent to which binge eating disorder should be considered a purely mental health disorder vs. a physiological/biological one. Weight regulation models of eating disorders are under development that propose weight and weight history are causal variables that have clinically significant impacts on eating disorder psychopathology and perpetuation (17). However, these models remain to be tested.

Here, we present findings from a mixed-methods, cross-sectional survey aimed to collect information from experts in the field about clinical aspects of adult binge eating disorder pathology.

## Methods

### Participants and recruitment

This study recruited expert researchers, clinicians, and healthcare administrators in the field of adult binge eating disorder. Eligibility criteria is previously published in Bray et al. (18, 19) and is shown in Table 1.

**Table 1:** Participant eligibility criteria.

***I. Eligibility criteria for researchers (18 recruited, 7 enrolled)***
**Eligibility criteria for researchers required meeting one of the following four criteria:**
1. ≥1 active R01, T32, or P grant on binge eating or food addiction **as i**dentified on NIH RePORTER (https://report.nih.gov)
2. Last author of ≥10 PubMed-indexed publications published 2010–2020 on adult binge eating disorder AND ≥5 PubMed-indexed publications in 2015–2020 on the same topic
3. Last author of ≥5 PubMed publications published in 2015–2020 relevant to food addiction^a^
4. Referral from someone who meets one of the qualifications above (I.1–3)
***II. Eligibility criteria for clinicians and healthcare***
***administrators (18 recruited, 6 enrolled)***
**Eligibility for clinicians and healthcare administrators required meeting** **≥3 of the following criteria:**
1. Award Winner or Honoree of the Association of Eating Disorders (AED, 2010–2020) or the Castle Connolly Top Doctors Distinction in Psychiatry—Eating Disorders (2020/21) (20, 21)
2. Executive position/board member for one of ten relevant societies: Academy of Nutrition & Dietetics, Academy of Eating Disorders (AED, FAED), American Society for Metabolic and Bariatric Surgery (ASMBS), Behavioral Health Nutrition Society, Eating Disorder Research Society (EDRS), International Association of Eating Disorder Providers (IAEDP), Johns Hopkins 2020 Eating Disorders Conference, National Center of Excellence for Eating Disorders (NCEED), National Eating Disorder Association (NEDA), Obesity Society (22–32)
3. Adult binge eating disorder provider listed in the National Eating Disorder Association (NEDA)– or Alliance for Eating Disorders Awareness Provider Directories (33, 34) or associated with an eating disorder program or treatment center with ≥5 locations listed in the NEDA directory (33)
4. Popular press distinction (35, 36)
5. Referral from an individual meeting ≥2 qualifications above
6. Registered Dietician (RD) meeting ≥2 criteria above
***III. Additional Eligibility Criteria (2 recruited, 2 enrolled** ^*b*^ **)***
Individuals who met ≥1 academic/research criterion (I) and ≥1 clinical criterion (II) were also eligible.

^a^This criterion required ≥5 publications in the past 5 years because of the relative newness of the concept of food addiction.

^b^Both participants each met two academic/research criteria and two clinician/healthcare administrator criteria.

### Procedure

The procedure is described in Bray et al. (18, 19). With approval from the National University of Natural Medicine (NUNM) IRB (# HZ12120), BB sent eligible participants a scripted email study invitation. Consenting respondents were interviewed anonymously on Zoom (Zoom.com, last accessed May 19, 2022), with verbal consent obtained at the start of each interview. Interviews were recorded with participant consent. Recordings began after introductions, to protect participant anonymity. Most interviews were scheduled for 2 h, with abbreviated 30–60-min interviews conducted as needed. Interview questions pertaining to binge eating disorder pathology are shown in Table 2. Demographic information was collected at the end of each interview verbally or through follow-up email survey.

**Table 2:** Interview questions pertaining to clinical factors relevant to adult binge eating disorder pathology.

**Question**	***n* asked (*n*/14)**
1. Please describe your perspective on (or knowledge of) literature and research findings, current clinical guidelines, and your own personal experiences that relate to binge eating disorder pathology and treatment.	14 (100%)
2. How do you view the disorder in relation to the following possible aspects, and how important is it for treatment interventions to address these aspects (if at all)?	
a) Physical/Biological b) Cognitive/mental c) Emotional d) Spiritual e) Economic f) Social g) Cultural h) Other	14 (100%) 14 (100%) 14 (100%) 14 (100%) 11 (79%) 12 (86%) 12 (86%) 14 (100%)
3. Please describe your view on the following health factors as they relate to adult binge eating disorder pathology and treatment:	
a) Metabolic Disorder b) Obesity	12 (86%) 12 (86%)
4. Are there any other aspects of binge eating disorder pathology that you feel are important to address or discuss (that have not been addressed above)?	12 (86%)
5. Please describe your perspective on current research gaps that exist in the field of binge eating disorder.	14 (100%)

Results expressed as *n* (%). *n*, number participants asked. Percentages expressed as *n*/14 times 100.

### Data analysis

Interview recordings were transcribed. Transcripts were de-identified and then reviewed and qualitatively analyzed by BB and HZ (separately) for common themes using a reflexive thematic analysis approach (37). BB and HZ independently coded each interview. Themes were identified independently then discussed and finalized through reflexive engagement with the data (37). BB also analyzed transcripts quantitatively to identify the number of participants who expressed positive/supportive, negative/skeptical, or neutral perspectives on each identified theme. HZ and CB were consulted when quantitative analysis questions arose and for tiebreakers.

### Participant response rates and characteristics

**Figure 1:**
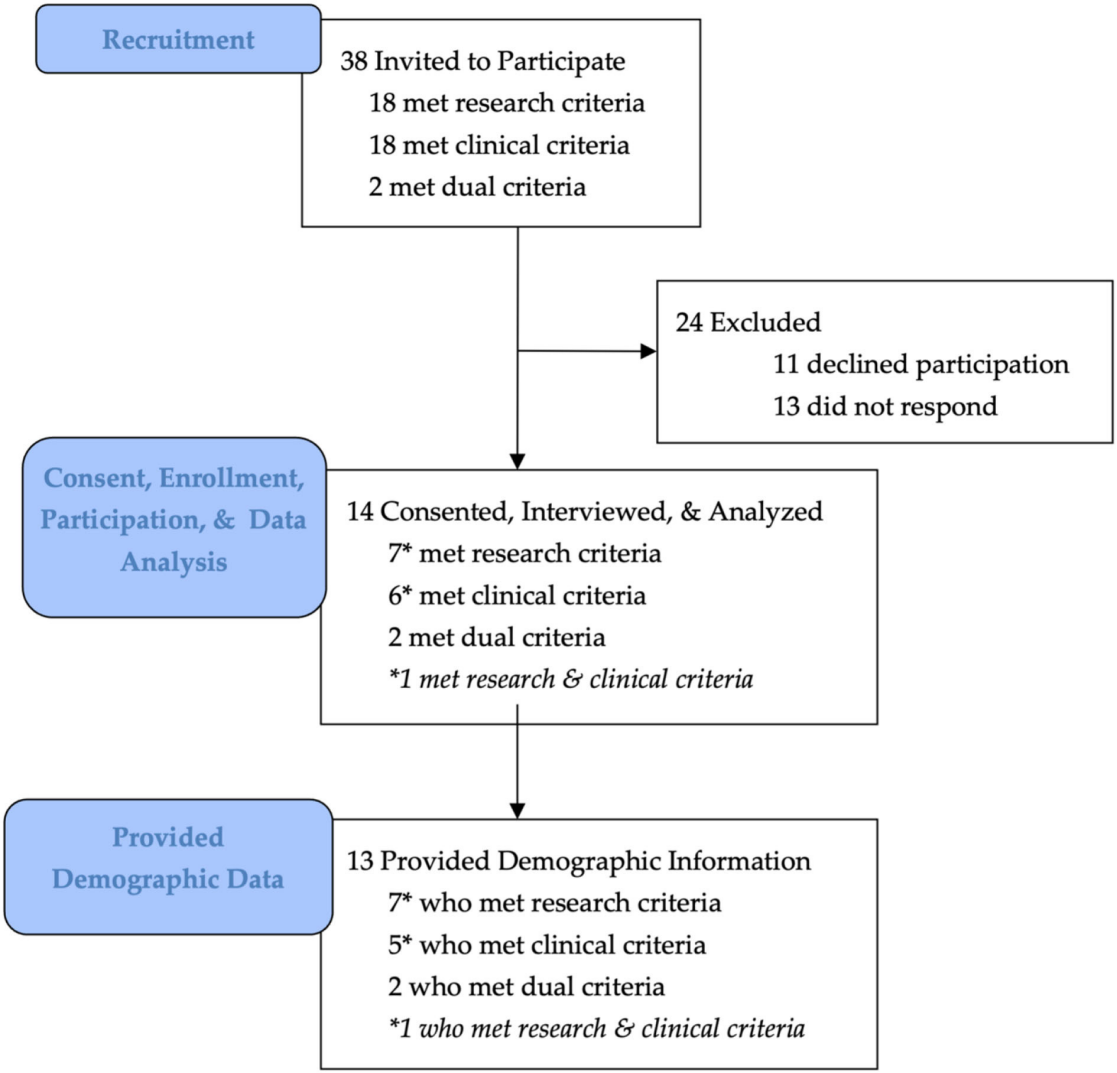
Diagram of study flow, from participant identification to enrollment and follow-up. Thirty-eight experts met enrollment criteria and were invited to participate in the study. This included 18 experts who met the academic/research criteria (18/38, 47%), 18 experts who met the clinical criteria (18/38, 47%), and two who met the dual criteria (2/38, 5%; Table 1). Fourteen eligible experts consented, enrolled, and participated in the study (14/38, 37%), including six individuals who met the academic/research criteria (6/14, 43%), five who met the clinical criteria (5/14, 36%), one who met both the academic/research and clinical criteria (1/14, 7%), and two who met the dual criteria option (2/14, 14%) (Table 3). Thirteen participants (13/14, 93%) provided demographic information and were included in demographic analysis (Table 3). All 14 participant interviews were included in thematic analysis. Reproduced with permission from Bray et. al., (18).

Thirty-eight experts met enrollment criteria and fourteen consented, enrolled, and participated in the study (Figure 1). Fourteen experts consented, enrolled, and participated in the study, including six individuals who met the academic/research criteria (6/14, 43%), five who met the clinical criteria (5/14, 36%), one who met both the academic/research and clinical criteria (1/14, 7%), and two who met some criteria from the academic- and clinical categories to qualify for inclusion in a mixed option (2/14, 14%) (Table 1). Table 3 shows characteristics for the 13/14 participants who provided demographic information.

**Table 3:** Characteristics of the 13/14 study participants who provided demographic data.

**Eligibility criteria met**	
Research/academic	6 (43%)
Clinical/administrative	5 (36%)
Both (research/academic and clinical/administrative)	1 (7%)
Combined (≥1 research/academic and ≥1 clinical administrative)	2 (14%)
**Accreditations**	
Fellow of the academy of eating disorders (FAED)	8 (62%)
Doctor of philosophy (Ph.D.) or science (ScD)	8 (62%)
Medical doctor (MD)	4 (31%)
Licensed or registered dietician (LD/RD) or registered dieticians certified in eating disorders (CEDRD)	4 (31%)
Healthcare administrator	2 (15%)
Certified chef	1 (8%)
Certified intuitive eating specialist (CIES)	1 (8%)
Fellow of the American college of neuropsychopharmacology (FACNP)	1 (8%)
Bachelor of medicine chirurgical doctor (bachelor of surgery) (B\MBChB)	1 (8%)
Masters in public health (MPH)	1 (8%)
**Sex assigned at birth**	
Female	8 (62%)
Male	5 (38%)
Other	0 (0%)
**Age**	
55 ± 10.2 years (range: 37–44 yrs., *n* = 13)	
**Ethnicity**	
Hispanic or Latino	0 (0%)
Not Hispanic or Latino	13 (100%)
**Race**	
American Indian or Alaska Native	0 (0%)
Asian	1 (8%)
Black or African American	0 (0%)
Native Hawaiian or Other Pacific Islander	0 (0%)
White	12 (92%)
More than one race	0 (0%)
**Geographical location of residence**	**7 reported**
United States of America (USA)	5 (71%)^**^
United Kingdom (UK)	1 (14%)^**^
Australia (AU)	1 (14%)^**^
Canada (CA)	1 (14%)^**^

Results expressed as n (%) or mean ± SD. Unless indicated, percentages are expressed as n/13 times 100, as one participant did not provide demographic data. ^**^Percentages are expressed as n/7 times 100, as only seven participants provided this data.

## Results

### Theme 1: obesity domain (100%)

All 14 participants (14/14, 100%) addressed the domain of obesity as relevant to binge eating disorder. Subthemes included: (i) the relationship between obesity and binge eating disorder (13/14, 93%); (ii) possible underlying mechanisms that link obesity to binge eating disorder (9/14, 64%); and (iii) validity of links to negative health consequences in binge eating disorder (4/14, 29%) (Table 4).

**Table 4:** Participant statements relating domain 1, “obesity domain” to binge eating disorder pathology (100%).

**Subtheme (i) relationship between obesity and BED**	**13 (93%)**
(a) Common link between obesity and BED	8 (57%)
(b) Many with BED struggle with obesity	5 (36%)
(c) Not everyone with BED has a larger body or obesity	4 (29%)
(d) Need for clarifying extent to which obesity and BED are separate vs. related/overlapping	4 (29%)
(e) Obesity can motivate BED treatment	3 (21%)
(f) Obesity as negative consequence of BED	2 (14%)
(g–j) Possible relationships identified by 1 participant each are included in Supplementary Table 1	
**Subtheme (ii) possible relationship mechanisms**	**11 (79%)**
(a) Contributions to weight stigma and resulting trauma^a^	6 (43%)^a^
(b) Inflammatory processes related to food choices and mood	4 (29%)
(c) Sleep disturbances	3 (21%)
(d) Links between obesity and the gut microbiome	2 (14%)
(e) Obesity impacting relationships and interpersonal factors that mediate/moderate binge eating	2 (14%)
(f–h) Possible mechanisms identified by 1 participant each are included in Supplementary Table 4	
**Subtheme (iii) validity of links to negative health consequences**	**4 (29%)**
(a) Obesity can increase risk for medical complications	2 (14%)
(b) Not everyone with obesity has negative health consequences^b^	2 (14%)
**Additional participant statements related to subtheme i, “relationship between obesity and BED”**	
“*Well **clearly lots of research has been done** on relationships of weight and high weight and binge eating disorder… that's where epidemiologists have done a lot” (P16)*	
**Additional participant statements related to subtheme iii, “validity of negative health consequences”**	
“*I see metabolic disorder as being one of the … scare tactics used when [addressing] an obesity epidemic: ‘look, [obesity is] associated with higher rates of metabolic disorder and high blood pressure,' and … [there are] certain things that are associated with, but there are definitely people with binge eating disorder, or people with obesity, who don't have any of those problems. … In other words, they don't have metabolic syndrome. They don't have high blood pressure, they don't have diabetes, and yet …obesity has been declared a disease like a disorder” (P38)*	
“*We do have this really strong assumption, and I think this weight stigma as well, that [is] shared by [some] physicians [but not all, and represents a] view in the general population that all overweight is unhealthy and that any degree of overweight must be bad for your physical health, and you must improve your physical health with any degree of weight loss. And that's just simply not true. I think that's weight stigma as well. …We need to address that” (P93)*	

Results expressed as n (%). Percentages: n/14 times 100. ^a^That are associated with weight and can contribute to- and/or exacerbate BED symptoms. ^b^E.g., metabolic disorder, diabetes, hypertension, and hypercholesterolemia. BED, binge eating disorder.

#### Subtheme i: Relationship between obesity and binge eating disorder (93%)

Thirteen participants made statements expressing views on the nature of the relationship between obesity and binge eating disorder pathology, including the possible directionality or statistical nature of the relationship (11/14, 79%). Eight participants endorsed a common link between obesity and binge eating disorder (8/14, 57%). Five participants (5/14, 36%) described obesity as a condition that many with binge eating disorder struggle with. Four participants (4/14, 29%) noted that not everyone with binge eating disorder has a larger body or obesity. Four participants endorsed a need for clarification on the extent to which obesity and binge eating disorder are separate vs. related/overlapping (4/14, 29%). Three participants expressed views that obesity can motivate treatment for binge eating (3/14, 21%). Two participants described obesity as a negative consequence of binge eating disorder (2/14, 14%). Additional possible relationships that were addressed by only one participant each are included in Supplementary material S1.1.

“**Many, many people with binge eating disorder have overweight or** ***obesity***.” (P5)

“*We interview people with obesity who say, I don't have binge eating, and then a lot of times they will self-report it.” (P72)*

“*We've struggled for a long time in the field with the **extent to which binge eating** **disorder and obesity are separate or related**, and like a lot of things in the mental health world, I think it's probably not so much an either-or place [but rather a] both-and place.” (P5)*

“*[Obesity is] **definitely relevant** [to binge eating disorder]. …It's one of those things that I think **everybody thinks that obesity and binge eating disorder have** … a one-to-one **[relationship]… [that] ‘everybody who is obese has binge eating disorder,'… which we know** **isn't true** but certainly, **obesity is**… one **of the most likely negative outcomes affiliated and** **associated with binge eating disorder** and …I think one of the things that most **motivates** **people to want to come in [for treatment]** because our society is really awful to people who have obesity and there's so much stigma that… both for the health consequences but also for the desire to have a different body shape is often what can get people in the door [for treatment].” (P19)*

“*People who self-identify as having binge eating disorder are – in my mind – a* **distinct** **subgroup from people with obesity** in that they experienced that sense of loss, loss of control, they're often **more distressed about their eating patterns**. They have more comorbidity. And there's certainly a lot of data that their healthcare utilization costs are higher. That may be psychiatric. I don't I don't think that's clear.” (P72)

#### Subtheme ii: Possible relationship mechanisms (79%)

Eleven participants (11/14, 79%) spontaneously described eight possible mechanisms by which obesity may be related to binge eating disorder. These included: (a) obesity contributing to weight stigma and resulting trauma that can contribute to and/or exacerbate binge eating disorder symptoms (6/14, 43%); (b) links to inflammatory processes related to food choices and mood (e.g., depression; 4/14, 29%); (c) obesity contributing to sleep disturbances that can exacerbate binge eating (3/14, 21%); (d) links between obesity and the gut microbiome (2/14, 14%); (e) obesity impacting relationships and interpersonal factors (e.g., isolation, social support, social anxiety) that mediate/moderate binge eating (2/14, 14%). Additional possible mechanisms addressed by one participant each are included in Supplementary material S1.2.

“There's so much **stigma**. That [the stigma] … – for the health consequences, but also for the desire to have a different body shape – is often what can get people in the door” (P19)

“**Stigma, …weight stigma… [and] obesity **…in some ways, that's what I think people with binge eating disorder are most concerned about.” (P38)

“I do think there are individuals whose bodies interact with things in our environment that might cause maybe **inflammatory responses**, or …disruption of the **gut biome** … where they might be **naturally inclined toward weight gain **– …maybe they are obese so they might be naturally inclined toward weight gain, or they have other medical issues that could make them inclined toward weight gain – so now you've got somebody who's got something going on inside of them, leading to weight gain that then **leads them to [food] restriction** that then leads them to binge eating.” (P7)

#### Subtheme iii: Validity of links to negative health consequences (29%)

Two participants (2/14, 14%) expressed views that obesity can increase risk for medical complications and two participants (2/14, 14%) stated that not everyone with obesity has negative health consequences (e.g., metabolic disorder, diabetes, hypertension, hypercholesterolemia).

“The cardiometabolic piece… it's just so easy to oversimplify it, right? To say, ‘well, **they're binge eating; therefore, they're overweight, therefore, they have** **cardiometabolic issues**.' I mean, what if it is bi-directional? And to what extent is this genetic? And to what extent are we looking at something that's much more complicated - is it **inflammatory?** Is it **mediated by the microbiome?** I don't know. But … we need to consider it because we need to look at it, **we need to make sure we're not stigmatizing our patients** **around it**. Obviously, it needs to be treated, you know, we need to be really, of course, looking out for **diabetes risk** but at the same time, you need to make sure you're **not putting** **people with a history of binge eating on too much of a restrictive diet**. I mean, if they end up being shamed around their eating, it just makes the whole situation worse.” (P72)

“[A study conducted in individuals with eating disorders and high weight that assessed] health status [found that participants'] **health status' [as a group] was quite good**. It wasn't impaired. [The study] measured their **cholesterol, weight, waist circumference, all the** **medical stuff**… [and found that] … [they were] a healthy group, even though technically they were labeled in the high weight category. …In fact, the physical health status [of the participants] at the beginning [of the study] was not impaired. …I think that's often forgotten, [that for] many people living with high weight …their physical health status is good or normal or not different… not abnormal …or however you want to describe it. …so, we need to get that message out. **There can be some people with high weight** **who do have a lot of medical problems, that's really true. … But [there are] also a** **lot of people with eating disorders with high weight who are very healthy**.” (P93)

### Theme 2: Intentional/voluntary or unintentional/involuntary restriction (100%)

All 14 participants spontaneously identified a relationship between binge eating disorder and food/eating restriction, whether voluntary (e.g., self-elected dieting) or involuntary (e.g., imposed by a parent, medical provider, or economic conditions) (14/14, 100%) (Table 5). Three subthemes were identified: (i) the relationship between restriction and binge eating (100%); (ii) spontaneously identified forms of restriction (43%); and (iii) factors contributing to restriction (43%).

**Table 5:** Participant statements consistent with theme 2, subtheme i, “relationship between restriction and BED” (100%).

**Subtheme (i) relationship between restriction and BED**	**14 (100%)**
Can or does lead to binge eating	13 (93%)
Expressed a personal view that restriction can/does lead to binge eating	11 (79%)
Described a perspective that restriction can/does lead to binge eating as being endorsed in the field, but did not endorse or negate the view personally	2 (14%)
Predominant phenotype of BED	3 (21%)
Perceive high prevalence of restriction among individuals with BED	2 (14%)
Restriction may index distress about weight or pre-existing BED^a^	2 (14%)
**Additional participant statements related to subtheme i, “relationship between restriction & BED”**	
“*There is a group without question that is just hardwired to be higher weighted, and they are big time restrictors” (P72)*	
“*About a third [of my clients] described dieting and want to stop that cycle, but it actually is binge eating disorder” (P37)*	

Results expressed as n (%), in which percentages are n/14 times 100.

^a^Rather than directly causing binge eating.

BED, binge eating disorder.

#### Subtheme i: Relationship between restriction and binge eating (100%)

All participant statements addressed the existence of a possible relationship between restriction and binge eating (14/14, 100%) (Table 5). Eleven participants expressed views that restriction can or does lead to binge eating (11/14, 79%). Two additional participants described this view as being endorsed by cognitive behavioral therapy and in the field but did not endorse or negate the view personally (2/14, 14%). Three participants described food restriction as a predominant phenotype of binge eating disorder (3/14, 21%). Two participants expressed perceptions that a high prevalence of restriction exists among individuals with binge eating disorder, whether the individuals themselves realize it or not (2/14, 14%). Two participants also expressed views that restriction may index distress about weight or pre-existing binge eating (rather than directly causing binge eating) (2/14, 14%). Select quotes from participants regarding the relationship between restriction and binge eating disorder are shown below and in Table 5.

“*The most common behavior that anybody does before they binge is they restrict, that's what they do before they binge.” (P7)*

“**Any restrictions on food will lead to more binges***.” (P37)*

“*[Research finds] that …**there's diversity in the phenotype** of binge eating disorder, with … one group [“about half”] …being more …restrictive-focused, and another group … having more issues with inhibitory control and cravings, and emotion dysregulation.” (P19)*

“*There's …a worry … in the eating disorder field that* **dieting causes binge eating***. A lot of that data, I think, comes from cross-sectional and follow-up studies, surveys of folks out in the world who were asked, ‘are you dieting?' and then, ‘are you binge eating?' or: ‘are you dieting now?' and they follow them up and sometime later, they're found to have an increased frequency of binge eating compared to folks who were not originally dieting*.

*…. The problem is it's **hard to know cause and effect** [when interpretating that data]. These epidemiological-type studies find associations, but **what you may be [indexing] with** **the dieting, …is distress about weight**, as opposed to real food restriction, caloric restriction. So … it's hard to be sure exactly how to interpret [the data]. Maybe one interpretation is, indeed, [that] the dieting led people to binge eat, but it's not the only interpretation. And the data from …controlled trials where people are put on a diet under some sort of medical, psychological, or nutritional supervision – **the** **evidence that … clinically overseen dieting produces binge eating – is slim-to-none**. …Now again …there are individuals who …certainly apparently cannot tolerate dieting without some real distress and behavioral disturbance, so I'm not suggesting anything otherwise. But as a general phenomenon**, I don't think it's been established that** **dieting … always leads to untoward consequences**.” (P46, both paragraphs above)*

#### Subtheme ii: Spontaneously identified forms of restriction (100%)

All participants (14/14, 100%) spontaneously identified different types of restriction, including (a) self-imposed dieting (11/14, 79%); (b) restriction coinciding with food scarcity or economic insecurity (9/14, 64%); (c) externally mandated (by a medical doctor or parents, often linked to weight) (2/14, 14%); and (d) restricting certain types of foods, food enjoyment, or calories (2/14, 14%) (Table 6). Select quotes from participants spontaneously identifying forms of restriction are shown below and in Table 6.

**Table 6:** Participant statements consistent with theme 2, subtheme ii, “forms of restriction” (100%).

**Subtheme (ii) forms of restriction identified by participants**	**14 (100%)**
(a) Dieting	11 (79%)
(b) Restricted food access resulting from economic precarity	9 (64%)
(c) Externally mandated (by medical doctor or parents)^a^	2 (14%)
(d) Restricting certain of foods, food enjoyment, or calories	2 (14%)
**Additional participant statements relating to subtheme ii, “forms of binge eating”**	
“*About a third [of my clients] described **dieting** and want to stop that cycle, but it actually is binge eating disorder” (P37)*	
“*I work with patients who have said, ‘well yeah, I have binge eating.' **I binge eat the first two weeks of the month ‘cause that's when we have food in the house and then there's no food in the house the last two weeks of the month.'** That's a systemic issue that I think needs to be addressed and needs to be talked about in terms of people's vulnerability to eating disorders” (P75)*	
“*When you put somebody on a **diet, it's a medical intervention**, … you're doing something physically to their body, and to their mind, so that's under the realm of …biological [interventions] because restriction and cutting someone's calories or cutting food groups or telling them to …count carbs, or keep points, or count calories, or whatever …that's really **external regulation**” (P7)*	

Results expressed as n (%), in which percentages are n/14 times 100.

^a^Often linked to weight.

BED, binge eating disorder.

“**Chronic dieting** is something that we are currently seeing more and more of where it becomes this **binge-restriction cycle***.” (P37)*

“*There is a group without question that is just* **hardwired to be higher weighted***, and they are* **big-time restrictors***.” (P72)*

“**Access to food** is a big, big deal. …In households where there's …**food scarcity***, [that] can lead to binge eating. You don't know when you're getting your next meal? And it's in front of you? And you're really, really, really hungry because you haven't eaten in a while. And then there's food around? What do any of us do when we're really hungry? We eat. Our brain[s]… – we – are in food-seeking mode, we're hungry, we're deprived, we're mentally deprived, we haven't enjoyed the pleasure of it, we're physically hungry, our body says to eat.” (P7)*

“*I've worked with hundreds of people, it feels like, who have a story that goes something like, ‘well, … when I was a kid, I had this big appetite and **my parents***, it really freaked them out, so they* **started to put me on a diet** or lock up the food or put me in Weight Watchers or whatever, because they were worried I would get fat,' and particularly … when genetically that person was just going to be a little bit larger-bodied anyway, that **fear of fatness was introduced at such an early age and** **connected to the limiting of food**, and we know that people who go on a diet are more likely to gain weight, so it's a self-perpetuating cycle that is really not helpful.” (P60)*

#### Subtheme iii: Underlying factors contributing to restriction (71%)

##### Foci a: Specific/micro factors (50%)

Seven participants spontaneously identified eight different factors contributing to restriction (7/14, 50%; Table 7), including: (a) body weight/shape/size (especially in naturally higher-weighted individuals, 4/14, 29%); (b) restricting to soothe or cope (2/14, 14%); and (c) shame around eating (2/14, 14%). Additional specific factors identified by one participant (1/14, 7%) each are included in Supplementary material S2.1.

**Table 7:** Participant statements consistent with theme 2, subtheme iii, “factors contributing to restriction” (71%).

**Foci (a) specific/micro factors**	**7 (50%)**
(a) Body weight/shape/size^a^	4 (29%)
(b) Restricting to soothe or cope	2 (14%)
(c) Shame around eating	2 (14%)
(d–h) Additional specific/micro factors identified by only 1 participant (7%) each are included in Supplementary Table 2	
**Foci (b) contextual/macro factors**	**5 (36%)**
(a) Culturally driven (linked to weight)	3 (21%)
(b) Socially reinforced	2 (14%)
(c) Biologically reinforcing	1 (7%)

Results expressed as n (%), in which percentages are n/14 times 100.

^a^Especially in higher-weighted individuals. See Supplementary Table 2 for additional results.

“*The actual* **restriction of food**…* deprivation, restriction, the mandate to not eat, the* **shame** that's induced at a very early stage in one's life related to **eating**, related to **hunger**, related to **body size***, that's an interpersonal experience that is tied in with our weight-obsessed society, where there's a culturally-driven mandate to be a body size and shape that oftentimes is incongruent with our …pre-determined natural body weight.” (P7)*

##### Foci b: Contextual/macro factors (36%)

At a macro level, restriction was described as often being culturally driven (linked to weight) (3/14, 21%), but also socially reinforced (2/14, 14%) and biologically reinforcing.

### Theme 3: Negative affect, distress, and emotion regulation (100%)

#### Subtheme i: Negative affect (100%)

##### Foci a: Negative affect addressed verbatim (50%)

Negative affect was addressed *verbatim* by seven participants (7/14, 50%; Table 8). Six participants described negative affect as driving binge eating (6/14, 43%). Three participants (3/14, 21%) described a mechanism by which binge eating is used to reduce or alleviate negative affect; two participants referenced literature supporting this possibility (2/14, 14%). Two participants suggested negative affect makes binge eating disorder and its associated symptoms more difficult to manage, in part through the added burden of managing binge eating disorder with a concurrent mood disorder. One participant (1/14, 7%) suggested negative affect can contribute to increased risk for binge eating disorder (and referenced work supporting this possibility).

**Table 8:** Participant statements consistent with theme 3, subtheme i, “negative affect”.

**Foci (a) negative affect when addressed verbatim**	**7 (50%)**
(a) Drives binge eating	6 (43%)
(b) Reduced or alleviated by binge eating	3 (21%)
Literature cited	2 (14%)
(c) Makes binge eating disorder and symptoms more difficult to manage^a^	2 (14%)
(d) Contributes to increased risk for binge eating disorder (work cited)	1 (7%)
**Foci (b) negative affective states**	**10 (71%)** ^b^
(a) Guilt	6 (43%)
(b) Shame	6 (43%)
(c) Poor self-esteem	5 (36%)
(d) Self-hate	2 (14%)
**Foci (c) mechanisms relating negative affective states to BED**	**5 (36%)**
(a) Negative affect states linked to eating behavior^c^	5 (36%)
Guilt	4 (29%)
Shame	3 (21%)
Self-esteem	1 (7%)
(b) Negative affect states linked to body image	2 (14%)
Shame	1 (7%)
Low self-esteem	1 (7%)
(c–f) Additional possible mechanisms that were identified by 1 participant (7%) each are included in Supplementary Table S3.1	
**Additional participant statements related to foci b, “negative affective states”**	
“*Negatively-associated valence mood[s]—things like **anger**, things like **anxiety**, things like **shame** [are very important aspects of binge eating disorder]” (P84)*	
“*[Speaking to] the guilt of [the] eating disorder behavior: [an individual with binge eating disorder may] feel like **it's all their fault** [and have] … cognitive thoughts like, **‘…I'll never be able to do this. This is all my fault. I have no willpower. I'm a terrible person. How come I can't do this,'**…. [these] cognitive aspects [of binge eating disorder] are of course, **influenced by environmental aspects**. People think things in part because they hear them in the environment and believe that they should think them, so the thoughts about ‘**I should be a certain way,' or, ‘I shouldn't eat this certain way,' or, ‘I should have control,' or, ‘I should have willpower,' or, ‘that person is judging me for ABC and I'm judging myself for XYZ,'** that cognitive thought pattern is so debilitating for so many people who then [think], ‘…I should be able to do something with that, with my eating or my whatever.' Not to mention the [deeper] thoughts that some of those [initial negative thoughts] connect to, of**, ‘I can't do anything,'** you know, the pieces that we target with **cognitive restructuring**, that all-or-nothing thinking, the … **disaster-framing, [the] sort of mystical … crystal ball thinking**—that they know what's happening… [Those cognitive processes are] **in large part based on environmental input and this sort of biological mismatch of, ‘I feel like I should be able to control this, but my body is so excited about that food that I can't, and now I feel like I failed.'** So that piece, I think is critical to help people to understand how their biology influences how they hear those messages, which influence how they think and how they feel and how they behave” (P60)*	
**Additional participant statements related to foci c, “underlying mechanisms”**	
“*We always think purely about what you feel”—sad, depressed, irritable, fill in the blank, some sort of negative affect—and thus you binge [eat or] overeat these ultra-processed foods. But I actually think there's another half of the loop that's been under-explored. So … when I think of tobacco, … it's like, ‘oh, you're stressed, you're irritable, you're anxious, [so] you smoked a cigarette'. But when you smoke a cigarette, you're going to have a crash, and it's going to put you into an irritable withdrawn state a couple hours after it, which is going to make you more prone to negative affect, which is going to make you more likely to want another cigarette, and it's this very dangerous, cyclical process. So, the cigarette itself drives forward a tendency to experience negative affect. I think with food, we're in the zone of just [thinking that] negative affect triggers overeating of food, but [we're not yet asking] ‘do these foods then lead …a couple hours later… to [feeling] more prone to experiencing negative affect?”' (P19)*	
“*I think … the conceptualization [that difficulties with negative affect play a role in risk for binge eating] would still sort of cycle back to … the question[s] of ‘why does this person have high levels of negative affect?' Or ‘why does this person have difficulty—when encountering high levels of negative affect—with managing that?' And it's very tempting … to draw lines back to environmental and genetic factors to help explain that” (P5)*	

Results expressed as *n* (%), in which percentages are n/12 times 100, since 12 participants addressed this theme.

^a^In part through the added burden of managing a concurrent mood disorder.

^b^Six participants did not use the term “negative affect” directly; four did.

^c^Including binge eating and loss of control.

BED, binge eating disorder.

“*A lot of … work has looked at the role of emotional or affective factors. …we …see, for example, we think that **difficulties with negative affect play a role in risk for binge eating** at some level. I think … the conceptualization would still sort of cycle back to … the question[s] of ‘**why does this person have high levels of negative affect?**' Or ‘**why does this person have** **difficulty – when encountering high levels of negative affect – with managing that**?' And it's very tempting … to **draw lines back to environmental and genetic factors** to help explain that.” (P5)*

“*We know that negative effect is often a **driver for binge eating** and …not just in terms of onset of eating disorder, [but] also [in terms of] **managing this disorder with a concurrent** **mood disorder**.” (P93)*

##### Foci b: negative affective states (71%)

Ten (10/14, 71%) provided descriptions of negative affective states (10/14, 71%; Table 8). These included: (a) guilt (6/14, 43%), (b) shame (6/14, 43%), (c) poor self-esteem (5/14, 36%), and (d) self-hate (2/14, 14%).

“*I've had a client …literally **ask me if God hates** **her**.” (P37)*

“*We know that one of the things that is so ubiquitous with binge eating disorder patients is just the amount of **guilt and shame** that they are carrying around with them, … the… **constant feedback loop** of **‘I can't believe I did this; I'm so ashamed of how much I ate;** **I'm ashamed of what I ate; I did this secretively in my car; I don't want anybody to know.'** I've worked with patients who have spent just huge amounts of money on food that they don't have and that adds to the **shame and the guilt** that we see with these episodes. … I think **the** **amount of … emotional and cognitive burden that these folks are carrying around can't be** **understated.”** (P75)*

“*…I think …if you couple [the cognitive burden of guilt and shame described in P75 quote above] with also living in a higher-weighted body – which many individuals with binge eating disorder have – there is an **additional burden of weight stigma** that not only do they **face from the outside world, but they also internalize**, so maybe they **beat themselves up for living in a larger body** and think, ‘**see, you're just** **doing this to yourself because you're engaging in these binge eating episodes**,' and certainly we know that **shame is not a good motivator for behavior change**, so they just get **stuck in these cycles that I think are really pernicious”** (P75)*

##### Foci c: Underlying mechanisms (36%)

Five participants (5/14, 36%) identified mechanisms relating negative affective states to binge eating disorder (Table 8). These included: (a) binge eating behavior being linked to negative affective states (5/14, 36%), including guilt (4/14, 29%), shame (3/14, 21%), and self-esteem (1/14, 7%); (b) binge eating behavior being linked to body image (2/14, 14%), including shame and low self-esteem (1/14, 7% each); and (c) binge eating driving negative affect through induction of subsequent withdrawal [e.g., opponent-process theory (38, 39)]. Additional possible underlying mechanisms spontaneously identified by one participant each (1/14, 7% each) are included in Supplementary material S3.1.1 and Supplementary Table S3.1.

“*There's no question – [based on] the ecological momentary assessment literature – that **negative affect tends to rise prior to the onset of the binge** **eating episode** and then [there's] some debate about it, but **based on the data, I** **think [binge eating] really does work well in the moment to reduce negative affect** **and to increase positive affect** and it looks like we're trying to map all this on to neurobiology.” (P72)*

“*I do think that there's conssiderable empirical support for the idea that reward models are helpful. Whether they're brain based or just experiential reward, the idea that – in some fashion – **the binge eating experience either reduces negativity, or perhaps induces a** **brief period of positivity in terms of emotional state**, I think that has empirical support.” (P33)*

“*I think …if you couple [the emotional and cognitive burden that these folks are carrying around] with also **living in a higher-weighted body **– which many individuals with binge eating disorder have – there is an **additional burden of** **weight stigma** that not only do they **face from the outside world, but they also** **internalize**, so maybe they **beat themselves up for living in a larger body** and think, ‘**see,** **you're just doing this to yourself because you're engaging in these binge eating** **episodes**,' and certainly we know that **shame is not a good motivator for behavior** **change**, so they just get **stuck in these cycles that I think are really pernicious.”** (P75)*

#### Subtheme ii: Distress (64%)

Distress was addressed by nine participants (9/14, 64%) (Table 9). Five participants described distress as central to binge eating disorder pathology (5/14, 36%) and four described it as impacting binge eating disorder development (4/14, 29%). Three participants identified distress as central to self-identification and treatment-seeking for binge eating disorder (3/14, 21%). Three participants recognized distress a central DSM diagnostic construct (1) and three described distress as a key criterion that differentiates individuals with binge eating disorder from those with overweight, obesity, or loss of control eating (3/14, 21% each).

**Table 9:** Participant responses consistent with theme 3, subtheme ii, “distress”.

**Subtheme (ii) distress**	**9 (64%)**
(a) Central to binge eating disorder pathology	5 (36%)
(b) Impacting BED development	4 (29%)
(c) Central to BED self-identification and treatment-seeking	3 (21%)
(d) Central DSM diagnostic construct^a^	3 (21%)
(e) Key criterion differentiating BED from overweight, obesity, or loss of control eating	3 (21%)
**Additional participant statements related to theme 3, subtheme ii, “distress”**	
“*There's **distress** around the binge eating that can be emotional but I think [it is] **really core**…” (P93)*	
“*People who self-identify as having binge eating disorder are—in my mind—a **distinct subgroup from people with obesity** in that they experienced that sense of loss of control, they're often **more distressed about their eating patterns**. They have more comorbidity. And there's certainly a lot of data that their healthcare utilization costs are higher. That may be psychiatric. I don't I don't think that's clear” (P72)*	
“*Binge eating disorder, … is **defined** really solely on the behavior of binge eating and the second criteria of diagnostic specifiers, and the third criteria is marked **distress** regarding binge eating” (P93)*	

Results expressed as n (%), in which percentages are n/12 times 100, since 12 participants addressed this theme.

^a^APA (1).

BED, binge eating disorder; DSM, diagnostic and statistical manual of mental disorders.

“*The main problem is this **distress regulation** in the first place.” (P53)*

“*Clearly, folks with binge eating disorder – or at least those folks who show up for treatment – have increased anxiety, increased depression*, **increased distress***, … a lot of guilt, and have certainly over-concern with [body] shape and weight, compared to similarly sized peers.” (P46)*

“*One of the **DSM criteria** basically calls for distress – **extreme distress **– about the behavior [of binge eating] and so these folks – certainly, on average – are quite distressed, psychologically, more than their peers, and including distress about the behavior. So … I think [binge eating disorder is characterized] primarily [by] those two components … a behavioral component [and a] **psychological component**.” (P46)*

“*There's probably **a detection piece when we're asking people to self-identify**, and my more cynical friends… [would say], ‘I really think some of this is self-identification that's linked with psychopathology. **If you're in a higher level of distress, you're** **more likely to say, 'yes, I can't stop eating.'** Whereas …if we observe people in laboratories, I bet you a lot of people who say, 'nah, I could stop,'…they [actually] cannot resist that next piece of pizza, if we have them in a blind… setting.“ (P72)*

#### Theme iii: Emotion regulation and negative urgency (64%)

Nine participants identified emotional regulation or negative urgency as being central to binge eating disorder pathology (9/14, 57%; Table 10). Two participants referenced empirical support (2/14, 14%). Six participants described a paradigm in which binge eating is used as a strategy for regulating, stabilizing, or coping with emotions or negative affect (6/14, 43%). One participant discussed emotion regulation as being related to food- and serotonin dysregulation (1/14, 7%). One participant questioned the impact of emotion regulation on binge eating disorder pathology, stating emotion regulation interventions have not been found to differ in their effectiveness from guided self-help cognitive behavioral therapy, suggesting the pathology may be equal parts emotional and cognitive behavioral. Additional findings are described in the Supplementary material S3.2 and Supplementary Table S3.2.

**Table 10:** Participant statements consistent with theme 3, subtheme iii, “emotion regulation and negative urgency”.

**Subtheme (iii) Emotional regulation & negative urgency**	**8 (57%)**
(a) Central to BED pathology	8 (57%)
(b) Described paradigm in which binge eating is used as a strategy for regulating, stabilizing, or coping with emotions or negative affect	6 (43%)
(b.1) Referenced empirical support	2 (14%)
(c–d) Additional statements related to subtheme iii, “emotion regulation and negative urgency,” that were identified by 1 participant (1/14, 7%) each are included in Supplementary Table S3.2	
**Additional participant statements related to subtheme iii, “emotion regulation & negative urgency”**	
“*The main problem is this distress regulation in the first place” (P53)*	
“*There are also issues around serotonin levels that can have impacts on cognitive abilities and emotional abilities. …I think [this is] a very strong biological thing that needs understanding. … [emotional aspects of binge eating disorder are] very important indeed; partly because the emotions go haywire when serotonin levels [get] low, so … when somebody tries to clean carbohydrates out of their diet, they're already in trouble, but also partly because of the mood-stabilization effects [of binge-eating]—people learn that binge [eating works] as a way of stabilizing mood against a background of invalidating environments, uncontrollable mood states, etc. [especially] negatively-associated valence mood, things like anger, anxiety, shame… ” (P84)*	

Results expressed as n (%), in which percentages are n/12 times 100, since 12 participants addressed this theme. BED, binge eating disorder.

“*I'm very interested in **negative urgency**, … I think of it as **inhibitory control times** **negative affect, [that causes an individual to] become extremely impulsive**. When [an individual is] in a negative affective state, [s/he feels] this urgency to get rid of [that state] and …will do it with food or alcohol or whatever is available… [so] **that emotional piece and** **finding alternative ways to regulate emotions **– I think – is really important because so much of it – like **eating to cope **– just seems like such a **key … transdiagnostic construct** that I think is very proximal to the actual use. [For example], if you're in a negative affect, but you're not feeling very impulsive, you might not act [on binge eating]. If you're impulsive, but you're in a very … stable, emotional state, you might not act [on binge eating]. It is that combination of ‘**I can't tolerate this,'** [so] a little distress tolerance in it, too.” (P19)*

“*[There's been work] done [showing binge eating] is strongly linked with **momentary** **emotion**. I don't think that's necessarily the only causal factor, but I think, certainly, **emotion** **dysregulation** is a big part of it…” (P72)*

“*It certainly seems to **play out in the trait-based literature** so far that people who struggle with bulimia and binge eating sort of tend to be a little bit more **impulsive and** **dysregulated**.” (P60)*

### Theme 4: Diagnostic heterogeneity and validity (71%)

Ten participants (10/14, 71%) expressed views related to the diagnostic validity and/or heterogeneity of binge eating disorder (Table 11).

**Table 11:** Participant statements related to theme 4, “diagnostic heterogeneity and validity”.

**Subtheme (i) Diagnostic heterogeneity**	**10 (71%)**
a) Possible binge eating disorder subsets or phenotypes spontaneously identified or referenced by more than one participant	9 (64%)
1. “Food/eating addiction” or reward-based phenotype^a^	4 (29%)
2. Trauma, adversity, or PTSD-like factors present and predominant	4 (29%)
3. ADD/ADHD-like presentations^b^	3 (21%)
4. Chronic dieting/restriction-mediated	3 (21%)
5. Obsession and/or compulsion around food and/or eating^c^	3 (21%)
6. Hyper-sensitivity (to taste, facial cues, or social threat)	2 (14%)
7. Mood or emotion dysregulation-driven	2 (14%)
8–19. Additional possible subsets of phenotypes that were spontaneously identified or referenced by only one participant (1/14, 7%) each are included in Supplementary Table S4.1	
(b) Proposed AN, BN, and BED each contain the same three subgroups^d^	1 (7%)
**Subtheme (ii) diagnostic validity**	**5 (36%)**
(a) Skepticism of the current diagnostic criteria	5 (36%)
(b) “Debate about how to measure binge size”	3 (21%)
(c) Questioned validity of binge eating disorder as a psychiatric disorder	2 (14%)
(d) Proposed re-classification of eating disorder diagnoses with recurrent binge eating (e.g., BED, BN)^e^	1 (7%)
**Additional participant statements related to subtheme ii, “diagnostic validity”**	
“*I wonder if people could binge eat without visible consequences, if it would be a problem at all” (P38)*	
“*And there's reasons to raise questions, to what degree is eating behavior of folks with binge eating disorder really different from the eating behavior of folks without binge eating disorder in the real world? Until somebody comes up with a **way of assessing objectively [not] self-report**, … eating behavior in the real world, we're not going to be able to sort that out” (P46)*	
“*There's a lot of **debate about how you measure binge size**. [Binges] are discrete episodes that, … definitely involve sort of large amount of food that are unusual, as the DSM-5 would define it. But then you also see episodes in which there's a loss of control where it wouldn't necessarily be clinically large, but a lot of those episodes are still linked with people's perception of psychopathology” (P72)*	
“*… I think going forward, we'll need to re-think, at some point, this dichotomy we've got between the two forms of recurrent binge eating [e.g., bulimia nervosa and binge eating disorder] and [have] some reconciliation of them…” “We need to have [a] much **better understanding of how we classify and … diagnose** … the relationship between binge*	
*eating disorder and other disorders of recurrent binge eating in terms of how we conceptualize and classify the disorders, and is binge eating… really so distinct from bulimia nervosa and other eating disorders or not? [And what are] other ways of conceptualizing eating disorders …maybe more based on … the psychological understanding of disordered eating behaviors, such as degree of over-obsessionality, over-control or impulsiveness and under control.” (P93)*	
“*[In a 2009 publication on] the **validity of binge eating disorder**,[(40)] …comparing people who have binge eating disorder to weight-matched individuals without binge eating disorder… there were a very limited number of differences between those two groups. …And so, for me, we need to own the empirical support for the idea of binge eating disorder and what in fact it really is, and how does it differ from normality and how does it differ from overweight and obesity” (P33)*	

Results expressed as n (%), in which percentages are n/14 times 100.

^a^Driven by mechanisms implicit in substance-related and addictive disorders (e.g., hedonic/reward-based symptoms).

^b^Having issues with “inhibitory control,” “impulsivity,” and “craving” or “reward responsivity”.

^c^For example, obsessively thinking about food or compulsivity around eating food.

^d^One “traditionally… considered over-control [-driven, with] high obsessive-compulsive rates [and] high levels of perfectionism,” one with disordered eating but low psychopathology, and one with higher impulsivity.” This individual also emphasized the importance and difficulty of targeting heterogeneity in treatment.

^e^Based on similar subsets observed within the different diagnoses (e.g., “the degree of over-obsessionally [and] over-control, or impulsiveness and under-control” that underpin disordered eating behaviors).

Abbreviations: ADD, attention deficit disorder; ADHD, attention deficit hyperactive disorder; AN, anorexia nervosa; BED, binge eating disorder; BN, bulimia nervosa; PTSD, post-traumatic stress disorder.

#### Subtheme i: Diagnostic heterogeneity (71%)

Ten participants made statements related to heterogeneity within binge eating disorder (10/14, 71%). Eight participants (8/14, 57%) expressed views of binge eating disorder as a heterogenous diagnosis that may encompass several different subsets or phenotypes.

“*Is [binge eating disorder] all one homogeneous thing? Or is it or is it **heterogeneous**? …I think that in the realm of mental health, we're not in the spot that we are with, say, pneumonia, where … we can generally diagnose it now down to a **biologically relevant** **subgrouping**.”* (P5)

“*…One of the trickiest parts of binge eating … is **targeting … heterogeneity**.”* (P72)

Nine participants (9/14, 64%) spontaneously identified or referenced a total of nineteen possible phenotypes or subsets of binge eating disorder (Table 11; Supplementary Table S4.1). The five most commonly spontaneously endorsed phenotypes/subsets included individuals with: (1) hedonic/reward-based symptoms or driven by mechanisms implicit in substance-related and addictive disorders (SRADs) (e.g., a “food/eating addiction” phenotype) (4/14, 29%); (2) trauma, adversity, or post-traumatic stress disorder-like factors (4/14, 29%); (3) attention deficit disorder (ADD)/attention deficit hyperactive disorder (ADHD)-like presentations (having issues with “inhibitory control,” “impulsivity,” and “craving” or ”reward responsivity” (3/14, 21%); (4) chronic dieting or restricting (3/14, 21%); and (5) obsession and/or compulsion around food and/or eating [e.g., “obsessively thinking about food” or compulsivity around eating food (3/14, 21%)].

“*I think there's a subgroup of people [who] either because of their inhibitory mechanisms or the reward mechanisms … really struggle with [being able to eat their highest risk foods]. And they can probably do it in a restaurant, but… do we have to make them? … I don't think we understood **heterogeneity of reward response or inhibitory** **mechanisms** [when exposure to one's high-risk foods was the conventional training for treatment].”* (P72)

“*[Based on] some of the neurocognitive data around inhibitory control, some of the cognitive remediation work that's coming out, it's pretty clear there's a subgroup of people [who] probably meet the phenotype that would be similar to sort of an **ADD/ADHD** kind of presentation, where you generally see inhibitory issues or … potentially a reward responsivity…”* (P72)

One participant (1/14, 7%) identified three possible phenotypes or subgroups that cut across all eating disorders (one group with high levels of perfectionism, control, and obsessive-compulsive tendencies, one group with disordered eating but low psychopathology, and one group with higher impulsivity).

“*If you have a group of people with anorexia, a group of people with bulimia, a group of people with binge eating disorder [and] a group of people with obesity, it doesn't matter how you define it, you almost always end up with three **groups**: you end up with a group that is traditionally considered [as having] **over-control … high, obsessive compulsive rates, high levels of** **perfectionism**, you've got a group that has the eating disorder, and then pretty **low psychopathology**, and then you've got a group that [is] more **impulsive**.” (P72)*

#### Subtheme ii: Diagnostic validity (36%)

Five participants (5/14, 36%) expressed skepticism of- or limitations in the current diagnostic criteria for binge eating disorder. Three participants (3/14, 21%) addressed a “debate about how [to] measure binge size”. Two participants (2/14, 14%) questioned the validity of binge eating disorder as a psychiatric disorder, one referencing a publication that found “a very limited number of differences” between individuals who have binge eating disorder and weight-matched individuals with overweight and obesity but not binge eating disorder (40), and emphasizing the need for “[continued help in separating] binge eating disorder as an entity from overweight and obesity”. One participant suggested the need to consider diagnostic reconfiguration in light of possible subsets of underlying psychopathology that are shared across a variety of eating disorders (1/14, 7%).

“*One of the things I think about it is the continued support of [binge eating disorder] as a psychiatric disorder*. **Is it in fact, a** **disorder?**”* (P33)*

“**We don't have data on the actual eating behavior of people in the real world**.”* (P46)*

“*Sometimes they're eating what we would consider to be unusually large amounts of food and sometimes not, so there's a difference between … the objective … versus subjective binge eating episodes.” (P75)*

### Theme 5: Paradigm shifts in understanding binge eating disorder (43%)

#### Subtheme i: Anorexi-centric paradigm for understanding binge eating disorder (36%)

Five participants described an “anorexic-centric” paradigm that has historically been used for understanding binge eating disorder pathology, epidemiology, and treatment (5/14, 36%; Table 12).

**Table 12:** Participant responses consistent with theme 5 “paradigm shifts”.

**Subtheme (i) “Anorexia-centric” paradigm for understanding BED**	**5 (36%)**
(a) “Anorexia-centric” understanding of who can have an ED	4 (29%)
(b) Historical research focus on anorexia and bulimia nervosa	3 (21%)
**Subtheme (ii) paradigm shift in understanding drivers for BED**	**4 (29%)**
(a) Old focus on voluntary intentional food/eating restriction	2 (14%)
(c) Old focus on body weight/shape/size over-valuation and dissatisfaction	2 (14%)
(d) Newly included understanding of the role of emotion regulation	2 (14%)
(e–g) See Supplementary Table S5.1 for additional comments and foci identified by only one participant (7%) each.	

Results expressed as n (%), in which percentages are n/12 times 100, since 12 participants addressed this theme. BED, binge eating disorder; ED, Eating Disorder.

“*How we think about eating disorders is that … **anorexia was kind of the granddaddy**, … the thing we knew first, and then bulimia kind of grew out of that next, and … people used to refer to [it] as … **failed anorexia**… … and I would say, in part that, … binge eating disorder… was … thought of … initially [as being] **like bulimia, but without the** **purging**.” (P19)*

“*The construct [of binge eating disorder] was identified by Stunkard a long time ago* *(**41**)* *but as an actively studied construct, it's relatively new. …That means that people who've been doing this for a while almost invariably – if they're studying [binge eating disorder] – will have studied other [eating disorders], and **it's a limited number of people who I think could** **really argue that their entire career has been spent on [binge eating disorder exclusively]**.” (P5)* (41)

##### Foci a: Historical research focus on anorexia and bulimia nervosa (29%)

Four participants expressed views that eating disorder research has historically focused more on anorexia nervosa and bulimia nervosa vs. binge eating disorder (4/14, 29%; Table 12).

“***There's so much less research on binge eating disorder** than [on] anorexia nervosa or bulimia nervosa.” (P16)*

“*A lot of [research is done in] more intensive levels of care than outpatient, and people with binge eating disorder are not as much represented there as people who have anorexia or bulimia.” (P5)*

##### Foci b: “Anorexi-centric” understanding of who can have an eating disorder (21%)

Three participants described a historically “anorexi-centric” understanding of who can have an eating disorder (e.g., ascribing eating disorders to thin, white, affluent, cis-gendered neurotypical females) (2/14, 14%; Table 12).

“*So much of the eating disorder perspectives and history and things that we attend to are very female-focused, … and come out of … the female gender orientation. Because … I think … **anorexia [nervosa] kind of set the stage [for a current understanding of eating** **disorder pathology and epidemiology**], [and anorexia nervosa] is so dominantly female,” (P19)*.

“*We know that unfortunately eating disorders have been hampered by these **old** **stereotypes about who's affected**, and that leaves millions of people undetected with an eating disorder. … The number of people that I've seen and done evaluations on who are really surprised to learn that the way that they've been eating is actually considered disordered and that they have an eating disorder and I think that that's especially true for … any individuals that don't fit that **stereotypical mold of who has** **an eating disorder** … [which is] a **young, thin, cis-gendered, white woman**…” (P75)*

See statement from Participant 5 in Section 3.5.1.1 above.

“***There's so much less** **research on binge eating disorder** than [on] anorexia nervosa or bulimia nervosa.” (P16)*

#### Subtheme ii: Paradigm shift in understanding drivers for binge eating disorder (29%)

Four participants described a shift in our understanding—as a field—of the mechanisms that can drive binge eating disorder (4/14, 29%; Table 12). Participants described old paradigms as focusing on voluntary intentional food/eating restraint (e.g., intentional fasting, see Theme 5) (endorsed by 2/14, 14%) and body weight/shape/size over-valuation and dissatisfaction (endorsed by 2/14, 14%) as driving binge eating. Participants described new paradigms as focusing on the roles of emotion regulation (2/14, 14%), inhibitory control (1/14, 7%), interpersonal factors (1/14, 7%), mood (1/14, 7%), and reward (1/14, 7%). Additional findings within this subtheme are described in Supplementary material S5 and Supplementary Table S5.1.

“*I would say traditionally, when I'm talking to my colleagues in the eating disorder field… the dominant mechanisms that … have a tendency to be most thought of are **things like** **restraint and [body] shape and weight overvaluations** but there's starting to be a bigger push to … have a **more encompassing view on mechanisms like reward and** **inhibitory control and emotion regulation**, things like that. … I think in part because the restraint stuff wasn't necessarily panning out with binge eating disorder quite as well.” (P19)*

“*We've seen a **shift in the understanding of [binge eating disorder**] from [a historically] classic [view that] fasting leads to overeating [and] binge eating – which I think it's still valid, obviously – but I think there's been also **greater understanding of the role of** **emotions and mood and negative affect as [driving] binge eating**, and then we have that shift in our …understanding of people with mood intolerance and various forms of personality vulnerability – who we do see as well, quite often – and it's often that **the binge eating is a form of emotion regulation**, similar to other forms of emotion regulation, that [a patient] may present with, and I think that's led us to an understanding of different forms of psychological therapy [for binge eating disorder].” (P93)*

“*As a field … we neglect **social anxiety disorder** because **we tend to think it's just about** **weight and shape, self-consciousness, I think we under-diagnose this**. … we need to be looking specifically at Social Anxiety Disorder and I think based on Janet Treasurer's work, we're going to end up seeing that there's links in …sensitivity to social threat, … the extent to which that's causal, secondary to the eating disorder … **understanding where anxieties sort of intersect and [understanding the] neurocognitive** **process …especially around threat sensitivity… is going to be really helpful.” (P72)***

### Theme 6: Research gaps and future research directives (50%)

Two subthemes were identified regarding gaps in the literature and future research the experts would like to see closed related to the above topics: (i) Seven experts (7/14, 50%) identified a need for a better understanding of the relationship between binge eating disorder and overweight and/or obesity, including: (a) a need for clarification around the extent to which binge eating disorder and obesity are separate vs. related/overlapping (4/14, 29%); (b) greater clarification and understanding of how binge eating disorder differs from overweight and/or obesity (3/14, 21%); (c) what health risks and metabolic implications are associated with binge eating (2/14, 14%); and (d) prevalence of binge eating disorder in large and small body sizes (1/14, 7%) (Table 13). (ii) Three experts (3/14, 21%), identified classification issues as an area warranting further research, including: (a) whether binge eating disorder *is* a viable disorder (1/14, 7%); (b) understanding the eating behavior of individuals with binge eating disorder as it occurs in the real world (1/14, 7%); and (c) consideration of reclassification of binge eating disorder with other eating disorders of recurrent binge eating (e.g., bulimia nervosa and binge-purge-type anorexia nervosa) (1/14, 7%).

**Table 13:** Participant responses related to theme 8, “research gaps and future directives”.

**Subtheme (i) better understanding of the relationship between weight and BED**	**7 (50%)**
(a) Need for clarification around extent to which BED and obesity are separate vs. related/overlapping	4 (29%)
(b) Greater clarification and understanding of how BED differs from overweight/obesity	4 (29%)
(c) What health risks and metabolic implications are associated with BED?	2 (14%)
(d) Prevalence of BED in large and small body sizes	1 (7%)
**Subtheme (ii) classification issues**	**3 (21%)**
(a) Whether BED *is* a viable disorder	1 (7%)
(b) Understanding the eating behavior of individuals with BED as it occurs in the real world	1 (7%)
(c) Consideration of reclassification of BED with other EDs of recurrent binge eating (e.g., BN, B-P-type AN)	1 (7%)

Results expressed as n (%), in which percentages are n/12 times 100, since 12 participants addressed this theme. AN, anorexia nervosa; BED, binge eating disorder; BN, bulimia nervosa; B-P-type AN, binge-purge-type anorexia nervosa; ED, Eating Disorder.

## Discussion

### Novelty and innovation

To the authors' knowledge, our study is among the first to synthesize expert opinion on clinical factors pertaining to adult binge eating disorder pathology (and among the first to synthesize expert opinion on aspects of adult binge eating disorder in general). Synthesizing expert opinion isn't common in the binge-eating field. As such, this novel study that describes clinical factors pertaining to binge eating provides insights and expands upon several themes influencing the recognition of binge eating disorder, highlighting its heterogenous presentation and challenges in its clinical diagnosis, ultimately impacting management strategies. Exploring several themes and identifying novel viewpoints enables hypothesis-generating questions previously unexplored, or only explored within a limited capacity.

Most recently, a 2020 latent class analysis investigating potential sources of heterogeneity among 775 treatment-seeking adults with overweight or obesity and binge eating disorder identified two classes of individuals with binge eating disorder who differed in body image concerns, distress about binge eating, and depressive symptomatology (42). The number of binge eating episodes was also significantly different between the two classes; whereas body mass index (BMI) was not a significant covariate in most models. The findings led the authors to critique the way we currently define binge eating disorder diagnostically, as current features used for diagnosis fail to adequately explain presenting heterogeneity. The study suggests there appear to be distinct subgroups within binge eating disorder, which was exposed by at least one of the experts interviewed here.

The important findings of our study in addition to the existing literature highlight the ongoing evolution in our understanding of heterogeneity in binge eating disorder, refining its diagnostic criteria, and pursuit for suitable management strategies outside of the constructs already dominated by anorexia nervosa and bulimia.

### Relationship of findings to existing literature

#### Theme 1: Obesity domain (100%)

The experts' general recognition that obesity and binge eating disorder are commonly—but not always—linked (**theme 1, subtheme i**) aligns with current incidence and prevalence estimates (13–16), however, the nature of the relationship is less clear amongst interviewed experts. The experts' emphasis on the role of body/weight/shape stigmatization (theme 1, subtheme ii) seems to align with psychological contributions to intense concerns about body weight/shape/size overvaluation and heightened incentive for change (17). Evidence suggests comorbid obesity and binge eating disorder is associated with more severe and prevalent levels of mental health disorders and negative affect than those observed in individuals with obesity or binge eating disorder alone (43–46).

Meanwhile, findings on *physical* health outcomes associated with comorbid obesity and binge eating disorder seem less clear, as recognized by the experts (theme 1, subtheme iii). A small observational study published in 2009 found 66% of treatment-seeking individuals with binge eating disorder and obesity had metabolic syndrome, with men and whites having significantly higher rates than women and African Americans, respectively (47). However, in this study, neither self-reported frequency of binge eating, nor severity of eating disorder psychopathology significantly differed among individuals with- vs. without metabolic syndrome. More recently, a 2014 factor structure analysis of metabolic syndrome in 347 individuals with obesity and binge eating disorder found metabolic syndrome factors (e.g., obesity, glucose regulation, blood pressure, and lipids) do not significantly differ in individuals with binge eating disorder and obesity vs. those found in normative population studies (48). However, authors suggested “moderate attempts to regulate food intake may reduce the negative impact of obesity and binge eating pathology on metabolic function”, (48). Furthermore, a 2008 review questions the validity of using obesity as a diagnostic criterion for binge eating disorder as the distress and psychopathology associated with binge eating disorder are not primarily due to obesity (19).

#### Theme 2: Intentional/voluntary or unintentional/involuntary restriction (100%)

The experts' general views that food restriction can or does lead to binge eating (**theme 2, subtheme i**) aligns with that of cognitive behavioral therapy (CBT), the first-line therapeutic intervention for any eating disorder, including binge eating disorder (3, 5, 6), which posits that dieting behavior drives binge eating and results from overevaluation of eating and body weight/shape/size (3). However, CBT fails to produce longstanding remission in ~50% of individuals with binge eating disorder (49), suggesting possible limitations in this view that are supported in the literature (7, 50–52).

The relationship between dietary restraint and economic precarity has recently gained recognition in the field (18). Here, food scarcity was recognized as a common form of food restraint by most experts, second to dieting (theme 3, subtheme ii). This recognition aligns with findings from several studies conducted at a food pantry in San Antonio, TX between 2015 and 2016 (53–55). These studies found 90% of respondents had a clinically significant eating disorder (55), with eating disorder pathology severity significantly correlating with deliberately trying to limit food consumption or going >8 h without food consumption (*r* = *0.25, p* = 0.0001), which 52% of respondents reported (53). Reasons for food/eating restraint included lack of resources, SNAP/food stamps being insufficient, and emphasizing other family members receive access to food (53). More recent findings suggest binge eating disorder is 1.65 times more common in indivdiuals with food insecurity (8.6% prevalence vs. 5.2% prevlance in food-secure indivdiuals; *p* = 0.02) (56) and both food insecurity and/or receiving government assistance before age 18 are both associated with increased odds of having binge eating disorder (57, 58).

#### Theme 3: Negative affect, distress, and emotion regulation (100%)

In line with general expert recognition of the links between negative affect, distress, emotion regulation, negative urgency, and binge eating (**theme 3**), literature supports adult binge eating disorder linked to psychosocial dysfunction across a wide range of domains, including affect and emotion regulation (59). The majority experts' identification of negative affect, emotion dysregulation, and negative urgency as driving binge eating (**theme 3**) aligns with emotion/affect regulation models, which are well-supported in the literature (4). This recognition also aligns with a paradigm shift in the field from a historical tendency to attribute all eating disorders to overvaluation of eating behavior and/or body weight/shape size and resulting dietary restraint (e.g., dietary restraint and dual pathway models) (3, 4, 7) to a more encompassing view of binge eating disorder as a heterogenous disorder with multiple possible underlying mechanisms and room to accommodate multiple conceptual models (4, 18). This trend was also recognized by several experts (**theme 5**). Experts also reflect a belief that research investigating directionality of the associations between binge eating and negative affect, emotion dysregulation, and negative urgency is needed, as is reflected in the literature (59).

The concept of alexithymia is also one that warrants discussion alongside the topic of emotion regulation processes. Alexithymia is a subclinical phenomenon involving a lack of emotional awareness thought to result from difficulty in identifying and describing one's feelings and in distinguishing feelings from bodily sensations of emotional arousal [(60) as cited in (61)]. The involvement of alexithymia in anorexia nervosa and bulimia nervosa has been demonstrated in the literature (62). The involvement of alexithymia has also been documented in individuals with obesity (without eating disorder diagnoses), both by self-report (63) and implicit measure (64). Several evidence are also available in the literature indicating the role of alexithymia in binge eating disorder (62, 65). Interestingly, the concept of alexithymia was only addressed specifically by one participant in this study (P72, Table 10). This participant's statement captures the intertwined relationship between alexithymia and emotion regulation. Future research investigating possible relationships between alexithymia, emotion regulation, and negative affect and urgency in binge eating disorder would be both interesting and impactful.

#### Theme 4: Diagnostic heterogeneity and validity (71%)

The experts' recognition of heterogeneity in binge eating disorder aligns with the literature, in which upwards of seven different models of binge eating disorder conceptualization have empirical support (4). Interestingly, the possible binge eating disorder subsets or phenotypes spontaneously identified or referenced by the experts tend to align with various models/conceptualizations of binge eating disorder (Table 14).

**Table 14:** Binge eating disorder models/theories/conceptualizations supported in the literature and participant statements that align with each theory/model/conceptualization.

**BED model/theory/** **conceptualization supported in the literature (4)**	**Brief explanation of the model/theory**	**Identified as a theme among expert responses?**	**Expert recognition of BED phenotype(s) that align with this model (theme 4, subtheme i, S4.1, and Table 11)**	**Other expert views that align with this model/theory**	
Dietary restraint models	Dietary restraint viewed as a prospective risk factor for binge eating (66)	Yes (theme 2, theme 4, subtheme i)	“Chronic dieting-restriction-mediated subtype” identified by 3 experts (21%; theme 4, subtheme i, Supplementary material S4.1 and Table S4.1)	93% of experts expressed views that restriction can or does lead to binge eating (theme 2, Table 5)	
Dual pathway models	Body dissatisfaction viewed as leading to binge eating through restrained eating and negative affect (7)	Not directly, though see columns 5–6	N/A	29% of experts identified body weight/shape/size as a factor contributing to restriction and 14% of experts described restricting to soothe or cope (theme 2, subtheme iii, Table 7)	43% of experts described a paradigm in which binge eating is used as a strategy for regulating, stabilizing, or coping with emotions or negative affect, though not necessarily linked to body dissatisfaction (theme 3, subtheme iii, Table 10)
					14% of experts described an old focus on body weight/shape/size overvaluation/dissatisfaction and restriction as driving binge eating, and a new understanding of the role of emotion regulation as a driving factor (theme 5, Table 12)
Emotion/affect regulation models	View that negative emotions, moods, or affective experiences can create discomfort that is alleviated by binge eating, thus negatively reinforcing the behavior (4)	Yes (theme 3, theme 4, subtheme i)	“Mood or emotion dysregulation-driven” subtype identified by 2 experts (14%; theme 4, subtheme i, Supplementary material S4.1 and Table S4.1)	All experts identified associations between negative affect and binge eating (100% endorsement: theme 3, Table 8)	43% of experts described a paradigm in which binge eating is used as a strategy for regulating, stabilizing, or coping with emotions or negative affect, though not necessarily linked to body dissatisfaction (theme 3, subtheme iii, Table 10)
					14% of experts described an old focus on body weight/shape/size overvaluation/dissatisfaction and restriction as driving binge eating, and a new understanding of the role of emotion regulation as a driving factor (theme 5, Table 12)
Escape (disassociation) models	View that binge eating is used to alleviate negative affect associated with high self-awareness (related to pressures, threats, long-term concerns, and lasting consequences of experiences) and hyper-awareness of failings to meet high internal and external expectations [(67) as cited in (4)]	No, though see columns 5–6	N/A	Escape (disassociation) models were not identified as a theme across expert interviews. However, several experts' descriptions of negative affect, negative urgency, and emotion regulation align with the view that individuals with BED have high awareness of their failings to meet the expectations set for them by themselves or others (theme 3, subthemes i, iii; Tables 8, 10)	
Food addiction models	View that binge eating can result from the same psychopathology and behavior that occurs in substance-related and addictive disorders, but in relation to certain foods, food aspects, or eating behaviors (e.g., highly palatable, processed, or caloric foods; sugar; binge eating behavior)	This was identified as a theme that is addressed in a separate manuscript about expert perspectives on food/eating addiction	“Food/eating addiction' or reward-based phenotype” identified by 4 experts (29%; theme 4, subtheme i, Supplementary material S4.1 and Table S4.1)	Expert opinions on the concept of food/eating addition and reward-based phenotypes are addressed elsewhere	
Integrative cognitive-affective theory (ICAT) models	View that self-discrepancy (disparity between how an individual views the self vs. comparisons to standards related to an “ideal/ought” self) can lead to negative affect, which can precipitate and negatively reinforce binge eating (68)	No, though see columns 5–6	N/A	43% of experts expressed views that negative affect drives binge eating (theme 3, subtheme i). One expert linked negative affect and binge eating to discrepancy between self-views in comparison to self-standards (P60, Table 8)	
Interpersonal models	View that relationships can crucially impact self-esteem, anxiety, and psychopathology either positively or negatively, with interpersonal stressors promoting negative affect and low self-esteem that binge eating is used to alleviate	This was identified as a theme that is addressed in Bray et al. (18)	“Social-anxiety-driven” subtype identified by 1 expert (7%; theme 4, subtheme i, Supplementary material S4.1 and Table S4.1)	See findings reported in Bray et al. (18)	
Neurocognitive/ neurobiological models	Emphasizes the role of neurocognitive factors (e.g., executive functioning, inhibitory control, set shifting, and reward processing) in increasing risk for loss of control and binge eating	No However, many of these concepts are addressed in a separate manuscript about expert perspectives on food/eating addiction	“General cognitive deficits/sequential issues” subtype identified by 1 expert (7%; theme 4, subtheme i, Supplementary material S4.1 and Table S4.1)	Many of these concepts are addressed in two separate manuscripts about expert mental health aspects of BED (19) and perspectives on food/eating addiction (forthcoming)	
Perfectionism models	View that socially prescribed perfectionism—or the perception of it—promotes vulnerability to binge eating by increasing interpersonal discrepancies and decreasing interpersonal esteem (4)	No	N/A	One participant expressed a view that AN, BN, and BED each contain the same three subgroups of: (a) control-driven individuals with high levels of perfectionism and high obsessive-compulsive rates; (b) individuals with low ED psychopathology but disordered eating; and (c) individuals with higher impulsivity (theme 4, subtheme i, P72)	
Schema models	View that unmet emotional needs can result in long-standing patterns of maladaptive thinking, feeling, behaving, and coping that can maintain eating disorder pathology (69)	No, though see columns 4–5	“Trauma, adversity, or PTSD-like subtype” (endorsed by 29% of experts) “Learned emotional invalidation” subtype (endorsed by 7% of experts) “Invalidating environments” subtype (endorsed by 7% of experts)	Expert opinions on trauma, adversity, and PTSD are addressed in Bray et al. (18, 19)	
Transdiagnostic model	Expanded conceptualization based on the original cognitive-behavioral theory of bulimia nervosa (5, 70, 71) that suggests a dysfunctional scheme for evaluating the self—including overvaluation of body weight/shape/size and eating behavior and perfectionistic tendencies—result in low self-esteem that promote extreme and maladaptive weight control behaviors that prompt a cycle of dieting/weight loss and refractory binge eating (4, 5)	This was not directly identified as a theme, though see columns 4–5	“Chronic Dieting/Restriction-mediated” subtype (endorsed by 21% of experts) partly aligns with this model	Participant descriptions of negative affective states (theme 3, subtheme i, foci b) and possible mechanisms relating negative affective states to BED (theme 3, subtheme i, foci c) support a view that overvaluation of eating behavior contributes to poor self-esteem, which perpetuate binge eating behavior and psychopathology	
Weight regulation models	View weight and weight history as causal variables with clinically significant impacts on ED psychopathology and perpetuation (17)	Though expert statements aligning with weight regulation models was not identified as a theme, obesity was (theme 1)	N/A	Although multiple links were identified between obesity and BED in theme 1, none of the experts in this study identified obesity or weight history as impacting or perpetuating BED psychopathology when specifically addressing the topic of obesity (theme 1)	93% of experts expressed views that restriction can or does lead to binge eating (theme 2, Table 5)
				29% of experts identified body weight/shape/size as a factor contributing to restriction (theme 2, subtheme iii, Table 7)	In the theme of restriction (theme 2 subtheme ii), 2 experts (P37, P60) described a pathology in which a natural predisposition for being higher-weighted results in internally- or externally imposed food/eating restriction, which induces or perpetuates a binge-restriction cycle or ED psychopathology
Two participants also expressed views that (1) AN, BN, and BED each contain the same three subgroups of: (a) control-driven individuals with high obsessive-compulsive rates and high levels of perfectionism; (b) individuals with low ED psychopathology but disordered eating; and (c) individuals with higher impulsivity (P72) and (2) re-classification of eating disorder diagnoses with recurrent binge eating (e.g., BED, BN) should be considered based on similar subsets observed within the different diagnoses [e.g., “the degree of over-obsessionality [and] over-control, or impulsiveness and under-control” that underpin the ED behaviors (P93) (theme 4, subthemes i and ii)].					

Columns 1 and 2 provide information on 12 different models/theories/conceptualizations of binge eating disorder supported in the literature (4). Column three shows whether the theme/model/conceptualization was identified as a theme among expert responses. Column four shows whether experts identified a binge eating disorder subtype of phenotype in theme 4, subtheme i (Table 11; Supplementary material S4.1 and Table S4.1) that align with each theory/model/conceptualization. Columns 5–6 display other expert views that align with each model/theory/conceptualization. AN, anorexia nervosa; BED, binge eating disorder; BN, bulimia nervosa; ED, eating disorder; ICAT, integrative cognitive-affective theory; PTSD, post-traumatic stress disorder.

Recognizing, accepting, identifying, and classifying heterogeneity in binge eating disorder is an important step toward matching client heterogeneity to treatment modality, as has been done successfully in other disorders (72). Future research needs to address concerns quantifying binge episodes and confirm whether additional objective criteria for “binge size” aids diagnostic validity.

Fortunately, progress to this end is underway. For example, a 2020 latent class analysis investigating potential sources of heterogeneity among 775 treatment-seeking adults with overweight or obesity and binge eating disorder identified two classes of individuals with binge eating disorder who differed most distinctly across differences in body image concerns, distress about binge eating, and depressive symptomatology (42). The findings led the authors to critique the way we currently define binge eating disorder diagnostically, suggesting “many features currently used to define binge eating disorder (e.g., binge-eating frequency) are not helpful in explaining heterogeneity among individuals with [the] disorder. Instead, body image disturbances, which are not currently included as a part of the diagnostic classification system, appear to differentiate distinct subgroups of [these] individuals… Future research examining subgroups based on body image could be integral to resolving ongoing conflicting evidence related to the etiology and maintenance of binge eating disorder,” (42). These important findings represent the ongoing evolution in our understanding of heterogeneity in binge eating disorder, and our ongoing evolution in refining binge eating disorder as a diagnosis.

#### Theme 5: Paradigm shifts in understanding binge eating disorder (43%)

Despite advances in the field of binge eating disorder, over one-third of interviewed participants continue to ascribe to an “anorexi-centric” understanding of binge eating disorder pathology, epidemiology, and treatment (**theme 5, subtheme i)**. As described previously by Bray et al. (18), an overwhelming majority of individuals satisfying DSM criteria for binge eating disorder fail to achieve an accurate diagnosis and/or receive adequate treatment (18). Furthermore, several minority and/or marginalized populations have a greater prevalence of binge eating disorder than the predominating white, cis-gendered, and heterosexual female described within the context of the “anorexi-centric” paradigm (18, 73–78). This phenomenon may result from reduced recognition and screening for binge eating disorder in minority and marginalized populations, which may result in turn from an “anorexi-centric” understanding of binge eating disorder and can further reinforce that understanding.

Overall, experts' recognition of our growing awareness of who can have an eating disorder (**theme 5, subtheme I, foci b**), the ways binge eating disorder differs distinctly from anorexia nervosa and bulimia nervosa (**theme 5, subtheme i**), and the heterogeneity in binge eating disorder factors and psychopathology that exists beyond dieting attempts are reflective of this recognition in the literature. These paradigm shifts offer hope for greater diagnostic specificity and treatment outcomes for this significant national and global health problem.

### Clinical implications

Our results call to light the need for a better understanding of the relationship between binge eating disorder and overweight and/or obesity, including a need for clarification around: (1) the extent to which the two health issues are separate vs. related/overlapping; (2) the validity of alleged health risks and metabolic implications associated with binge eating; and (3) the prevalence of binge eating disorder among individuals in large and small body sizes (**theme 1, theme 6**).

Further, while most experts expressed views about binge eating disorder psychopathology that align with dietary restraint models and emotion dysregulation models, a minority of experts recognize a historic trend in the field to view binge eating disorder as an extension of anorexia nervosa and bulimia nervosa. The experts also recognize a shift in these old paradigms toward greater recognition of who can have an eating disorder and around the heterogeneity that exists within the binge eating disorder diagnosis. It is important for clinicians to remember that the anorexi-centric stereotype of who can have an eating disorder (e.g., thin, White, affluent, cis-gendered, neurotypical females with anorexia nervosa) is outdated, and that binge eating disorder has a uniquely higher prevalence among racial, ethnic, sexual, gender-, and socioeconomic minorities [as also identified in (18)]. Thus, our findings also underscore the importance of equal and adequate screening for binge eating disorder across race, ethnicity, sexual and gender orientation, body weight/shape/size, and socioeconomic status. It is also important to identify ways to include marginalized individuals who do not have access to adequate information, screening, or treatment in binge eating research and help find treatment interventions accessible to them [see (18, 79)].

Lastly, our findings underscore the need for ongoing research on heterogeneity among binge eating disorder and for ongoing discussion and investigation of the way in which we diagnose and classify binge eating disorder. Improving diagnostic accuracy and specificity can help improve treatment specificity and outcome measures in turn.

### Study limitations and strengths

Although it would have been interesting to analyze interview transcripts with the specific question of which theoretical and conceptual models of binge eating disorder were spontaneously endorsed by experts [such as those identified by (4)], to do so would contradict the open-ended methodology of reflexive thematic analysis. Thus, the authors did not analyze transcripts with any conceptual models in mind and were blind to any information on various conceptual models prior to their analyses of the interview transcripts. We feel that overall, this nuance makes the analysis both unique, innovative, and more accurate and informative in its findings.

The qualitative and reflexive nature of this analysis limit its reproducibility, as the themes identified by the researchers are subjective based on their independent and joint analyses. Furthermore, the qualitative analysis of expert interviews was conducted by two individuals (BB and HZ with aid of CB). Thus, we cannot assess how accurately the themes identified here represent the true themes valued by expert binge eating disorder researchers (including those in this study and those at large). However, limitations are standard in the field of reflexive thematic qualitative analysis and are not generally viewed as discounting the methodology as a whole (37).

As is also standard for most qualitative research, it is important to note the study's sample size (which is appropriate for a mixed methods analyses of this nature) limits the generalizability of the data's themes and conclusions to the field of binge eating disorder researchers and clinicians at large. Additionally, as has been pointed out in previous publications (18, 19), one of the study's four possible eligibility criteria for researchers were NIH grant funding (Table 1), which presents a bias for including participants from the U.S. There were three other nationally independent eligibility criteria researchers could meet to be included in this study and the final study sample included participants from the UK, AU, and CA as well as from the US. Nevertheless, 50% of participants were from the US and including criteria for funding form other federal agencies could have improved the population representation of the study overall. Additionally, this study collected demographic data on sex assigned at birth but not on gender. This unfortunate oversight follows an old convention (asking for sex assigned at birth rather than gender) that fails to account for equity and diversity inclusion and collects information that is not demographically relevant (sex assigned at birth) but misses information that is more demographically relevant (gender).

This study utilizes several methodological strengths that counterbalance the limitations identified above. The study's systematic inclusion criteria (Table 1) provides strong population representation of academic and clinical experts who lead the field. This includes researchers with the greatest recent and historic funding and publication records and clinicians with high clinical and academic engagement and access potential (e.g., those most likely to be identified through google searchers by individuals with binge eating disorder). Second, the study sample includes a well-rounded balance of binge eating disorder experts, including PhD/SciD researchers, medical doctors (MDs), licensed therapists and dieticians (LPs, RDs, LDs), and intuitive eating specialists, healthcare administrators, and public health advocates (MPHs) (Table 3).

## Conclusions

Overall, our understanding of adult binge eating disorder as an autonomous eating disorder diagnosis continues to grow and expand. Experts most commonly endorse food/eating restriction and emotion dysregulation as important components of binge eating disorder psychopathology, which aligns with two common historically supported models of binge eating disorder conceptualization (dietary restraint theory and emotion/affect regulation theory). At the same time, some experts recognize a historical oversight of viewing binge eating disorder through a limited “anorexi-centric lens”, particularly in relation to who is at risk for having an eating disorder and factors that drive binge eating. The experts identify several areas of binge eating disorder that continue to warrant further investigation. These include the extent to which binge eating disorder and obesity are separate vs. related/overlapping and improving our understanding of the heterogeneity that exists within the diagnosis. Overall, these results highlight the continual advancement of the field to better understand adult binge eating disorder as an autonomous eating disorder diagnosis.

## Data availability statement

The raw data supporting the conclusions of this article will be made available by the authors, without undue reservation.

## Ethics statement

The studies involving human participants were reviewed and approved by National University of Natural Medicine (NUNM) IRB (# HZ12120). Written informed consent for participation was not required for this study in accordance with the national legislation and the institutional requirements. Informed consent was obtained verbally without use of participant names, to protect participant anonymity.

## Author contributions

BB and HZ: conceptualization, methodology, formal analysis, investigation, and project administration. BB: resources, data curation, and writing—original draft preparation. BB, AS, CB, HZ, and RB: writing—review and editing. HZ and RB: supervision. All authors have read and agreed to the published version of the manuscript. All authors agree to be accountable for the content of the work.
