# Supplementary material for: Clinical aspects of binge eating disorder: A cross-sectional mixed-methods study of binge eating disorder experts' perspectives
Source: Front Psychiatry. 2023 Feb 14;13:1087165. doi: 10.3389/fpsyt.2022.1087165 (PMC9971930; doi:10.3389/fpsyt.2022.1087165)
Supplement: Supplementary file 1 [file Data_Sheet_1.pdf]

Clinical Aspects of Binge Eating Disorder: A Cross-Sectional Mixed-Methods Study of Binge Eating Disorder Expert's Perspectives

Supplementary Material

Brenna Bray[1*], Adam Sadowski,[1] Chris Bray[2], Ryan Bradley[1,3], Heather Zwickey[1]

[1]Helfgott Research Institute, National University of Natural Medicine, Portland, OR, USA
[2]Wilder Research Division, Amherst H. Wilder Foundation, Saint Paul, MN, USA
[3]Herbert Wertheim School of Public Health, University of California, San Diego, CA, USA

* Correspondence:
Brenna Bray
brenna.bray1@gmail.com

Clinical Aspects of Binge Eating Disorder: A Cross-Sectional Mixed-Methods Study of Binge Eating Disorder Expert's Perspectives

SUPPLEMENTAL MATERIAL

Supplementary Material S1. Theme 1: Obesity (100%)

S1.1. Subtheme i: Relationship between obesity and binge eating disorder (79%)

One participant stated it was not known whether binge eating disorder increases risk for obesity (1/14, 7%). One participant expressed a view that obesity is not causative for binge eating (1/14, 7%). One participant also described obesity as masking binge eating disorder, citing that an individual with binge eating disorder may be more likely to seek treatment for their weight than for their binge eating (1/14, 7%).

*"**I don't think obesity is a cause**." (P37)*

*"Many folks are going to be **more open to seeking treatment for obesity** and/or even depression/anxiety but not [binge eating disorder]. This might result in the [binge eating disorder] going undiagnosed and untreated for extended periods of time (which has bearing on prognosis)." (P75)*

S1.2. Subtheme ii: Possible Relationship Mechanisms (79%)

f) links between obesity, economic status, and food/nutrition access (1/14, 7%); **g)** links between obesity and gut hormones (1/14, 7%); and **h)** possible genetic links between obesity and binge eating disorder (1/14, 7%).

Table S1: Participant statements related to theme 1, "obesity domain" that were made by only one participant each

Subtheme i) Relationship between obesity and BED	***11 (79%)***
g) Not everyone with a larger body has BED	1 (7%)
h) Obesity is not causative for BED	1 (7%)
i) Obesity masking BED[1]	1 (7%)
j) Unknown whether BED increases risk for obesity	1 (7%)
Subtheme ii) Possible relationship mechanisms	***11 (79%)***
f) Links between obesity, economic status, and food/nutrition access	1 (7%)
g) Links to gut hormones	1 (7%)
h) Possible genetic links between obesity and binge eating disorder	1 (7%)
Subtheme iii) Validity of links to negative health consequences	***6 (12%)***
c) Not everyone with a larger body has BED	1 (7%)

Table Legend: Results expressed as n (%). Percentages: n/14 times 100. **Abbreviations: BED**, binge eating disorder.

S2. Theme 2: Intentional/Voluntary or Unintentional/Involuntary Restriction (100%)

S2.1. Subtheme iii: Factors contributing to restriction (43%)

S2.1.1. Foci a: Specific/Micro Factors (50%)

d) Caffeine use (1/14, 7%); **e)** disorder/disease management (e.g., restricting to manage symptoms of irritable bowel syndrome or diabetes mellitus) (1/14, 7%); **f)** loss of control eating (1/14, 7%); **g)** shame around hunger (1/14, 7%); and **h)** trauma (1/14, 7%).

> *"I think that* ***IBS [and] diabetes*** *[have] the same sort of impact: if somebody has diabetes, and has to manage their food intake differently, that can set them up for binge eating or restricting. ... I think any ... illness that has a strong nutrition component – meaning you have to change something about your eating to manage the illness – puts people at a higher risk for an eating disorder." (P60)*

Table S2: Participant statements related to theme 2, subtheme iii, foci a, "specific/micro factors" that were made by 1 participant (7%) each

Foci a) Specific/Micro Factors	***7 (50%)***
d) Caffeine use	1 (7%)
e) Disorder/disease management (e.g., IBS, DM)[1]	1 (7%)
f) Loss of control eating	1 (7%)
g) Shame around hunger	1 (7%)
h) Trauma	1 (7%)

Table Legend: Results expressed as n (%), in which percentages are n/14 times 100. [1]E.g., restricting to avoid symptoms of IBS. **Abbreviations: BED**, binge eating disorder; **DM**, diabetes mellitus; **IBS**, irritable bowel syndrome.

S3. Theme 3: Negative Affect, Distress, & Related Symptoms/States (100%)

S3.1. Subtheme i: Negative Affect (100%)

S3.1.1. Foci c: Underlying Mechanisms (36%)

Additional possible underlying mechanisms spontaneously identified by one participant (1/14, 7%) each included: **d)** Binge eating linked to general genetic and/or environmental factors; **e)** externally oriented shame around eating; **f)** generally presenting self-esteem issues; **g)** links to stigmatization; and **h)** low self-esteem increasing eating disorder vulnerability when linked to perfectionism (**Table S5.1**).

Table S3.1: Participant statements related to theme 3, subtheme ii, "negative affect" made by 1 participant (1/14, 7%) each

Foci c) Mechanisms relating negative affective states to BED	***5 (36%)***
c) Negative affect states linked to externally oriented shame around eating	1 (7%)
d) Negative affect states linked to stigmatization	1 (7%)
e) Negative affect states linked to generally presenting self-esteem issues	1 (7%)
f) Low self-esteem increasing eating disorder vulnerability when linked to perfectionism	1 (7%)

Table Legend: Results expressed as n (%), in which percentages are n/12 times 100, since 12 participants addressed this theme. **Abbreviations: BED**, binge eating disorder.

S3.2. Subtheme iii: Emotion Regulation and Negative Urgency (64%)

One participant discussed emotion regulation as being related to food- and serotonin dysregulation (1/14, 7%). One participant questioned the impact of emotion regulation on binge eating disorder pathology, stating emotion regulation interventions have not been found to differ in their effectiveness from guided self-help cognitive behavioral therapy **Table S3.2**).

> *"...some people would say [binge eating disorder is] an emotion regulation problem; well then how come CBT [cognitive behavioral therapy] guided self-help works equally well to emotion regulation treatment?" (P33)*

Table S3.2: Participant statements related to theme 3, subtheme iii, "emotion regulation and

negative urgency” made by 1 participant (7%) each

c) Emotion regulation related to food- and serotonin dysregulation	1 (7%)
d) Questioned the impact of emotion regulation on BED pathology[1]	1 (7%)

Table Legend: Results expressed as n (%), in which percentages are n/12 times 100, since 12 participants addressed this theme. [1] Stating emotion regulation interventions have not been found to differ in their effectiveness from guided self-help cognitive behavioral therapy; see “additional participant statement” from P33 in section S4.3. **Abbreviations: BED**, binge eating disorder.

S4. Theme 4: Diagnostic Heterogeneity and Validity (71%)

S4.1. Subtheme i: Diagnostic Heterogeneity (100%).

In addition to the spontaneously identified or referenced binge eating disorder subsets or phenotypes identified in the main text, one participant each (1/14, 7%) each identified 11 additional possible subsets or phenotypes, which are shown in **Table S4.1**.

Table S4.1: Participant statements related to theme 4.1, "diagnostic heterogeneity" made by 1 participant (7%) each

Subtheme i) Diagnostic heterogeneity	***10 (71%)***
a) Additional possible binge eating disorder subsets or phenotypes that were spontaneously identified or recognized by one participant (1/14, 7%) each:	9 (64%)
8. Childhood/developmental environment factors	1 (7%)
9. Comorbid night eating syndrome	1 (7%)
10. Depression-mediated	1 (7%)
11. Economic/socio-economic status	1 (7%)
12. General cognitive deficits/sequential issues[1]	1 (7%)
13. Genetic Factors	1 (7%)
14. Invalidating environments	1 (7%)
15. Learned emotional invalidation	1 (7%)
16. Non-specific gastrointestinal and/or inflammatory issues[2]	1 (7%)
17. Non-specific personality factors	1 (7%)
18. Motivated by feeling of volume	1 (7%)
19. Social anxiety-driven	1 (7%)

Table Legend: Results expressed as n (%), in which percentages are n/14 times 100. [1]E.g., difficulties with daily activities of living. [2]"That group with that funky, inflammatory, GI stuff, pain, eating disorder, depression, anxiety, they have that whole horrible mix, most of them [also] have a trauma history." **Abbreviations: ADD**, attention deficit disorder; **ADHD**, attention deficit hyperactive disorder; **AN,** anorexia nervosa; **BED**, binge eating disorder; **BN**, bulimia nervosa; **PTSD**, post-traumatic stress disorder.

S5. Theme 5: Paradigm Shifts in Understanding Binge Eating Disorder (43%)

S5.1. Subtheme ii: Paradigm Shift in Understanding Drivers for Binge Eating Disorder (29%)

In addition to the shifts in our understanding – as a field – of the mechanisms that can drive binge eating disorder that were recognized by more than one participant each, one participant (1/14, 7%) each described new paradigms as focusing on the roles of inhibitory control (1/14, 7%), interpersonal factors (1/14, 7%), mood (1/14, 7%), and reward (1/14, 7%) (**Table S5.1**).

Table S1: Foci related to Theme 5, Subtheme ii, “Paradigm Shift in Understanding Drivers for Binge Eating Disorder” that were identified by only one participant (1/14, 7%) each

e) Newly included understanding of the role of mood	1 (7%)
f) Newly included understanding of the role of reward	1 (7%)
g) Newly included understanding of the role of inhibitory control	1 (7%)

Table Legend: Results expressed as n (%), in which percentages are n/12 times 100, since 12 participants addressed this theme. **Abbreviations: BED**, binge eating disorder; **ED**, Eating Disorder.
